# Supplementary material for: Omicron-specific mRNA vaccination alone and as a heterologous booster against SARS-CoV-2
Source: Nat Commun. 2022 Jun 6;13:3250. doi: 10.1038/s41467-022-30878-4 (PMC9169595; doi:10.1038/s41467-022-30878-4)
Supplement: Supplementary file 1 — Supplementary Information [file 41467_2022_30878_MOESM1_ESM.pdf]

Supplementary information

Fang et al.

Omicron-specific mRNA vaccination alone and as a heterologous booster against SARS-CoV-2

Inventory of supporting information

Supplementary figures and legends

Supplementary source data and statistics

Provided in excel file, “Omicron_ELISA_neutralization_summary.xlsx”.

List of oligos

Supplementary figures and legends

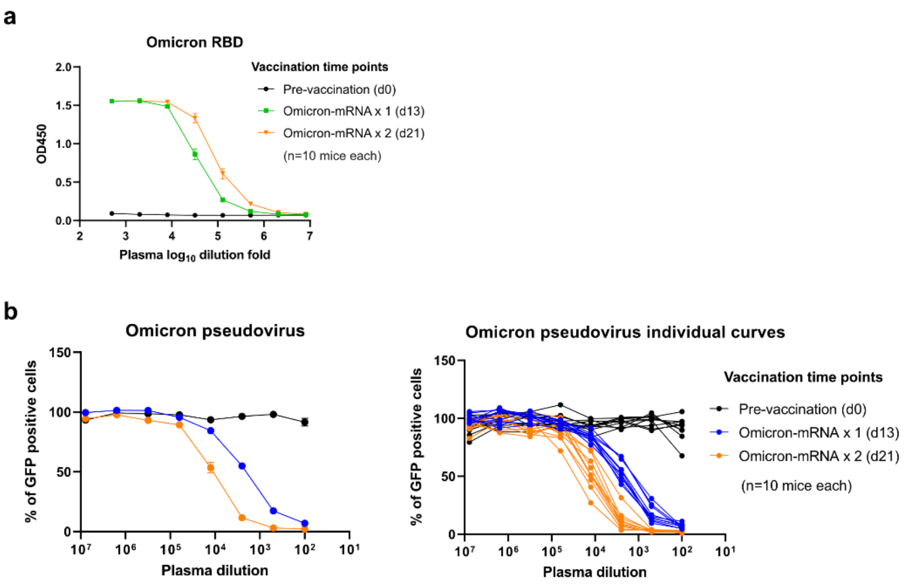

Supplementary Figure 1. ELISA and neutralization titration curves over serial dilution of plasma collected at different timepoints from mice administered with PBS or WT and/or Omicron LNP-mRNA.

a, ELISA titration curves over serial $\log_{10}$-transformed dilution points of plasma collected from mice before and after immunization with Omicron LNP-mRNA at defined time points (n = 10). Average curves, data are shown as mean ± s.e.m..

b, Omicron pseudovirus titration curves over serial $\log_{10}$-transformed dilution points of plasma collected from mice before and after immunization with Omicron LNP-mRNA at defined time points (n = 10). Left panel, average curves, data are shown as mean ± s.e.m.; Right panel, individual curves.

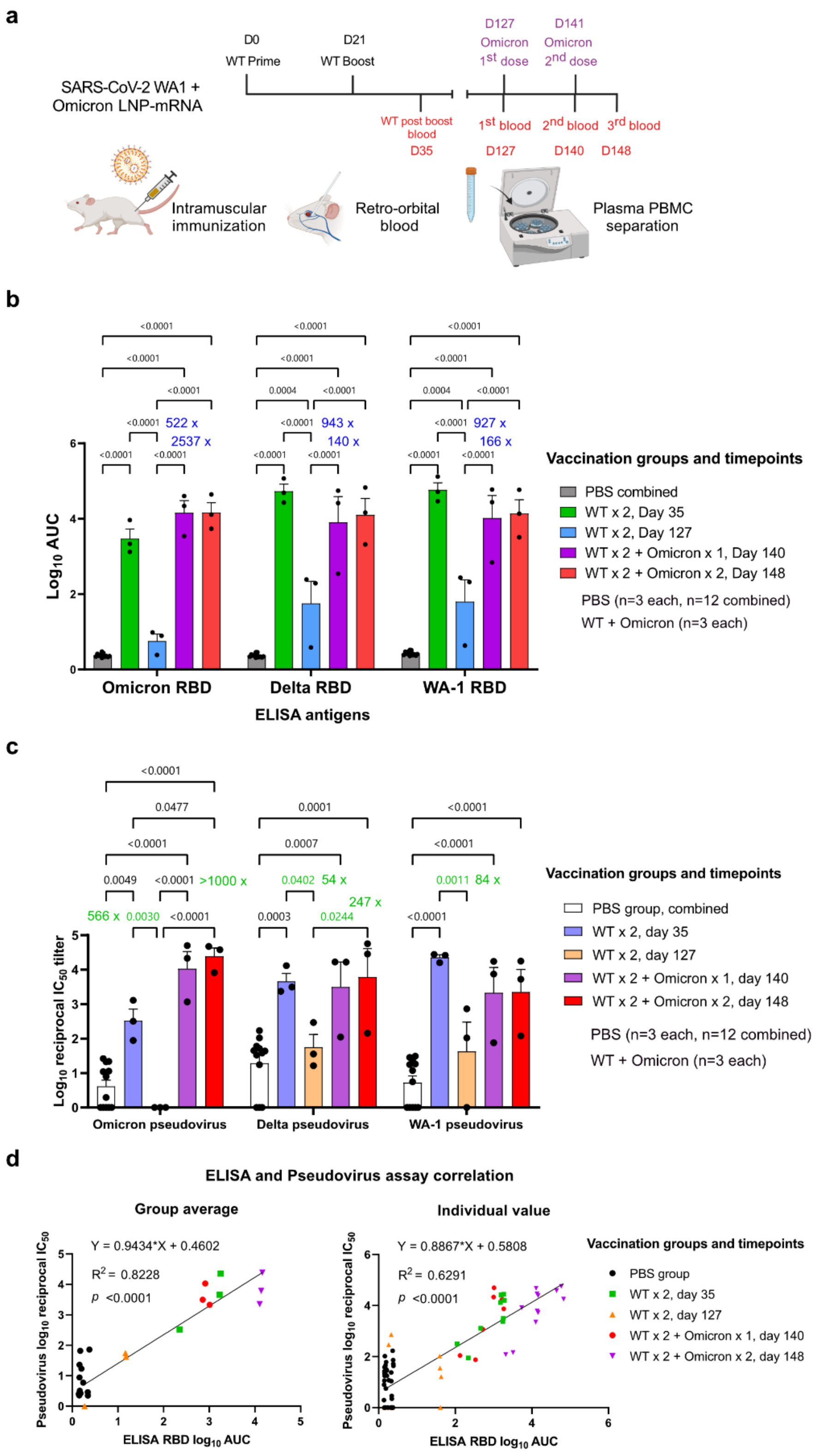
a
D0
WT Prime
D21
WT Boost
D127
Omicron
1st dose
D141
Omicron
2nd dose
SARS-CoV-2 WA1 +
Omicron LNP-mRNA
WT post boost blood
D35
1st blood
D127
2nd blood
D140
3rd blood
D148
Intramuscular immunization
Retro-orbital blood
Plasma PBMC separation
b
Log10 AUC
Omicron RBD
Delta RBD
WA-1 RBD
ELISA antigens
522 x
2537 x
943 x
140 x
927 x
166 x
Vaccination groups and timepoints
PBS combined
WT x 2, Day 35
WT x 2, Day 127
WT x 2 + Omicron x 1, Day 140
WT x 2 + Omicron x 2, Day 148
PBS (n=3 each, n=12 combined)
WT + Omicron (n=3 each)
c
Log10 reciprocal IC50 titer
Omicron pseudovirus
Delta pseudovirus
WA-1 pseudovirus
566 x
>1000 x
54 x
247 x
84 x
Vaccination groups and timepoints
PBS group, combined
WT x 2, day 35
WT x 2, day 127
WT x 2 + Omicron x 1, day 140
WT x 2 + Omicron x 2, day 148
PBS (n=3 each, n=12 combined)
WT + Omicron (n=3 each)
d
ELISA and Pseudovirus assay correlation
Group average
Y = 0.9434*X + 0.4602
R2 = 0.8228
p <0.0001
Individual value
Y = 0.8867*X + 0.5808
R2 = 0.6291
p <0.0001
Pseudovirus log10 reciprocal IC50
ELISA RBD log10 AUC
Vaccination groups and timepoints
PBS group
WT x 2, day 35
WT x 2, day 127
WT x 2 + Omicron x 1, day 140
WT x 2 + Omicron x 2, day 148

Supplementary Figure 2. Both WT and Omicron specific LNP-mRNA booster shots greatly improved waning immunity of mice vaccinated with SARS-CoV-2 WT LNP-mRNA against SARS-CoV-2 Delta and Omicron variants (Independent experiment 1 or batch 1).

a, Schematics showing the immunization and blood sampling schedule of mice administered with 1 µg WT LNP-mRNA prime (WT x 1) and boost (WT x 2) as well as 10 µg WT or Omicron-specific LNP-mRNA booster shots. The plasma and PBMCs were separate from blood for downstream assays. Created with BioRender.com

b, Bar graph comparing binding antibody titers of mice administered with PBS or WT and Omicron LNP-mRNA against Omicron, Delta and WT RBD (ELISA antigens). The antibody titers were quantified as Log_{10} AUC based on titrations curves in Extended Data Figure 1a. PBS sub-groups (n=3 each) collected from different matched time points showed no statistical differences between each other, and were combined as one group (n=9).

c, Neutralizing antibody titers in the form of log_{10}-transformed reciprocal IC50 calculated from fitting the titration curve with a logistic regression model (n = 3).

d, Correlation of neutralization titers (log_{10} reciprocal IC50, y axis) and ELISA titers (log_{10} AUC, x axis) from matched vaccination group (left panel) or individual mouse (right panel). PBS samples from different timepoints were shown as one group in correlation map and were not included in linear regression model. Each dot in bar graphs represents value from one group average (left panel), or one individual mouse (right panel).

Titer ratios were indicated in each graph and fold change is calculated from (ratio - 1). Data on dot-bar plots are shown as mean ± s.e.m. with individual data points in plots. Two-way ANOVA with Tukey's multiple comparisons test was used to assess statistical significance. Statistical significance labels: * $p < 0.05$; ** $p < 0.01$; *** $p < 0.001$; **** $p < 0.0001$. Non-significant comparisons are not shown, unless otherwise noted as n.s., not significant.

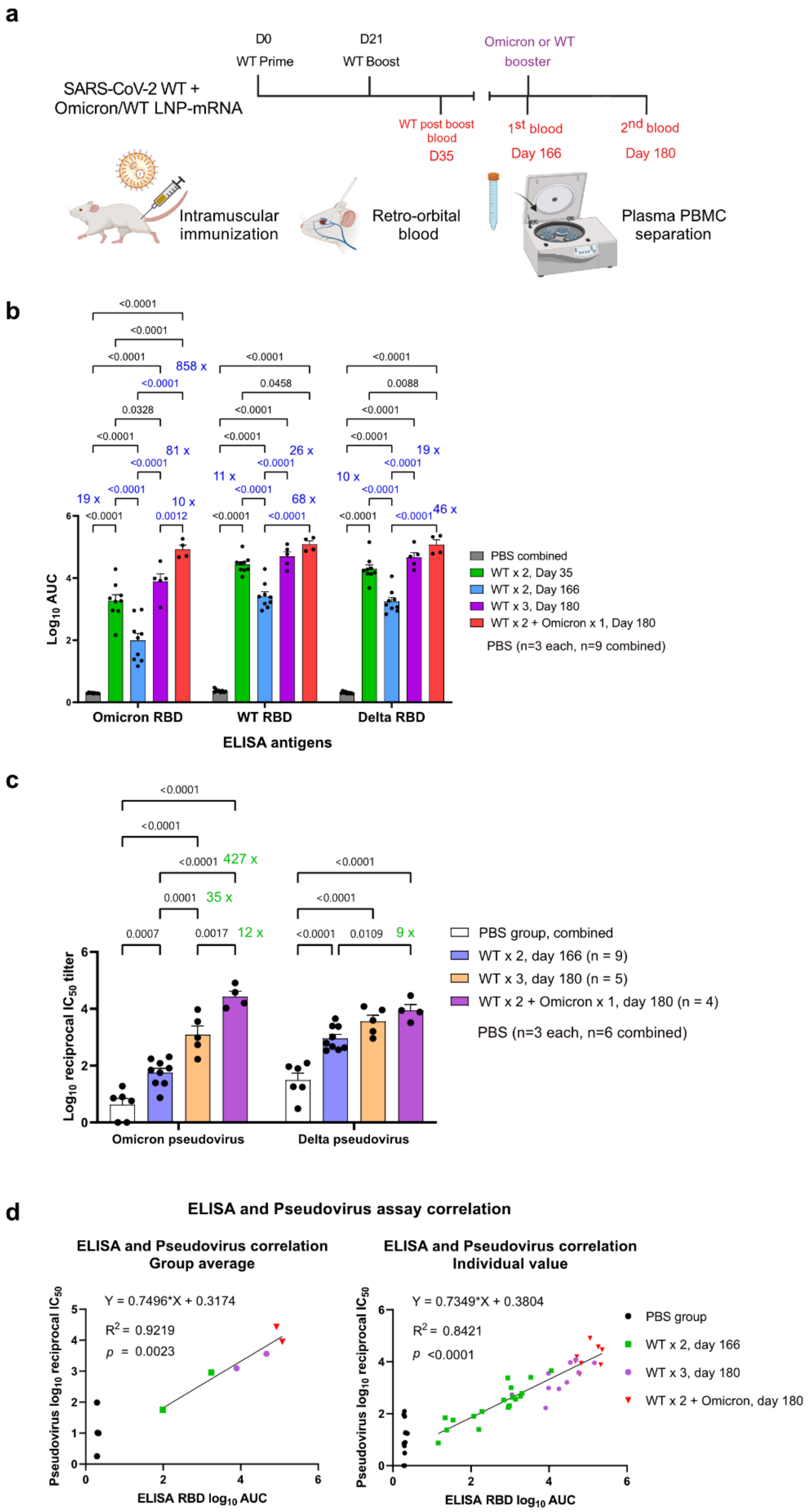
a
D0
WT Prime
D21
WT Boost
Omicron or WT booster
SARS-CoV-2 WT + Omicron/WT LNP-mRNA
WT post boost blood
D35
1st blood
Day 166
2nd blood
Day 180
Intramuscular immunization
Retro-orbital blood
Plasma PBMC separation
b
Log10 AUC
Omicron RBD
WT RBD
Delta RBD
ELISA antigens
PBS combined
WT x 2, Day 35
WT x 2, Day 166
WT x 3, Day 180
WT x 2 + Omicron x 1, Day 180
PBS (n=3 each, n=9 combined)
858 x
81 x
19 x
10 x
26 x
11 x
68 x
19 x
10 x
46 x
c
Log10 reciprocal IC50 tilter
Omicron pseudovirus
Delta pseudovirus
PBS group, combined
WT x 2, day 166 (n = 9)
WT x 3, day 180 (n = 5)
WT x 2 + Omicron x 1, day 180 (n = 4)
PBS (n=3 each, n=6 combined)
427 x
35 x
12 x
9 x
d
ELISA and Pseudovirus assay correlation
ELISA and Pseudovirus correlation Group average
ELISA and Pseudovirus correlation Individual value
Y = 0.7496*X + 0.3174
R2 = 0.9219
p = 0.0023
Y = 0.7349*X + 0.3804
R2 = 0.8421
p <0.0001
Pseudovirus log10 reciprocal IC50
ELISA RBD log10 AUC
PBS group
WT x 2, day 166
WT x 3, day 180
WT x 2 + Omicron, day 180

Supplementary Figure 3. Omicron specific LNP-mRNA booster shots greatly improved waning immunity of mice vaccinated with SARS-CoV-2 WT LNP-mRNA against SARS-CoV-2 Delta and Omicron variants (Independent experiment 2 or batch 2).

a, Schematics showing the immunization and blood sampling schedule of mice administered with 1 µg WT LNP-mRNA prime (WT x 1) and boost (WT x 2) as well as 10 µg Omicron-specific LNP-mRNA booster shots. The plasma and PBMCs were separate from blood for downstream assays. Created with BioRender.com

b, Bar graph comparing binding antibody titers of mice administered with PBS or WT and Omicron LNP-mRNA against Omicron, Delta and WT RBD (ELISA antigens). The antibody titers were quantified as Log_{10} AUC based on titrations curves in Extended Data Figure 1a. PBS sub-groups (n=3 each) collected from different matched time points showed no statistical differences between each other, and were combined as one group (n=6).

c, Neutralizing antibody titers in the form of log_{10}-transformed reciprocal IC50 calculated from fitting the titration curve with a logistic regression model (n = 9 before booster, n=5 in WT x 3, n = 4 in WT x 2 + Omicron).

d, Correlation of neutralization titers (log_{10} reciprocal IC50, y axis) and ELISA titers (log_{10} AUC, x axis) from matched vaccination group (left panel) or individual mouse (right panel). PBS samples from different timepoints were shown as one group in correlation map and were not included in linear regression model. Each dot in bar graphs represents value from one group average (left panel), or one individual mouse (right panel).

Titer ratios were indicated in each graph and fold change is calculated from (ratio - 1).

Data on dot-bar plots are shown as mean ± s.e.m. with individual data points in plots. Two-way ANOVA with Tukey's multiple comparisons test was used to assess statistical significance. Statistical significance labels: * $p < 0.05$; ** $p < 0.01$; *** $p < 0.001$; **** $p < 0.0001$. Non-significant comparisons are not shown, unless otherwise noted as n.s., not significant.

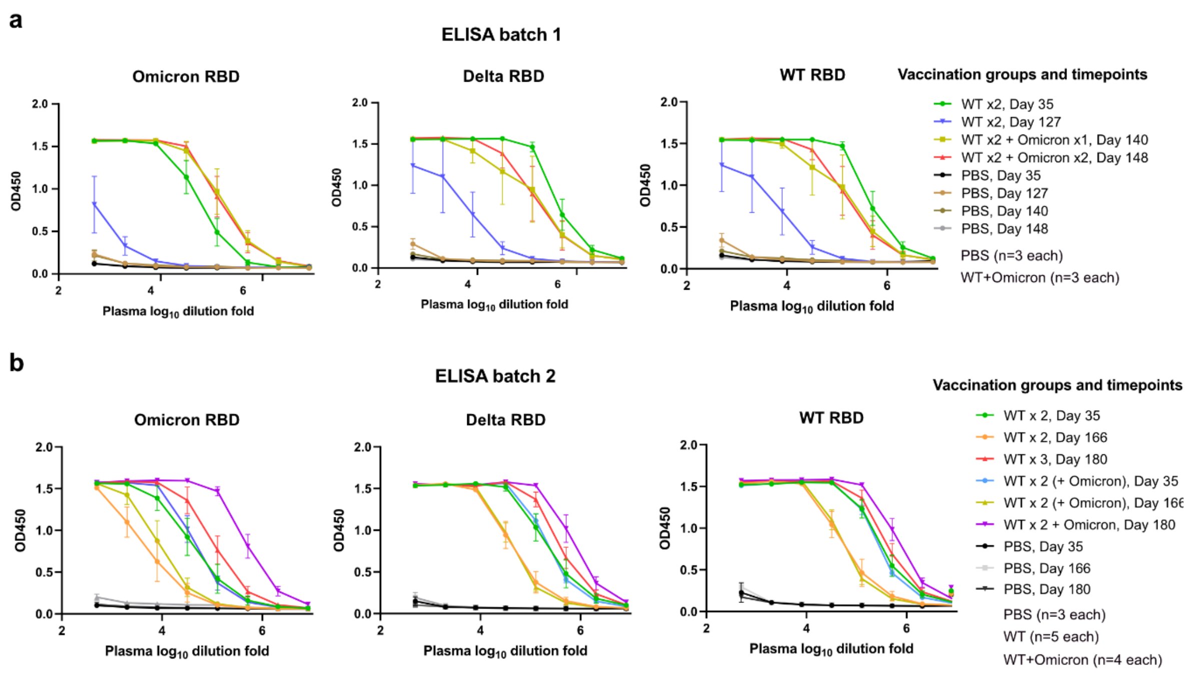

Supplementary Figure 4. ELISA titration curves over serial dilution of plasma collected at different timepoints from mice administered with PBS or WT and/or Omicron LNP-mRNA.

a, ELISA titration curves of batch 1 experiment (n = 3).

b. ELISA titration curves of batch 2 experiment (n = 9 before booster, n=5 in WT x 3, n = 4 in WT x 2 + Omicron).

The OD450 values were plotted against a series of log_{10}-transformed dilution points of plasma from mice 35 days post WT prime, >4 months post WT prime (day 127 in batch 1 and day 166 in batch 2) and 2 weeks post booster (day 140 in batch 1 and day 180 in batch 2) of WT or Omicron LNP-mRNA, against spike receptor binding domain (RBD) antigens of Omicron variant (left), Delta (mid) and WT (right) were shown. Data are shown as mean ± s.e.m. in plots.

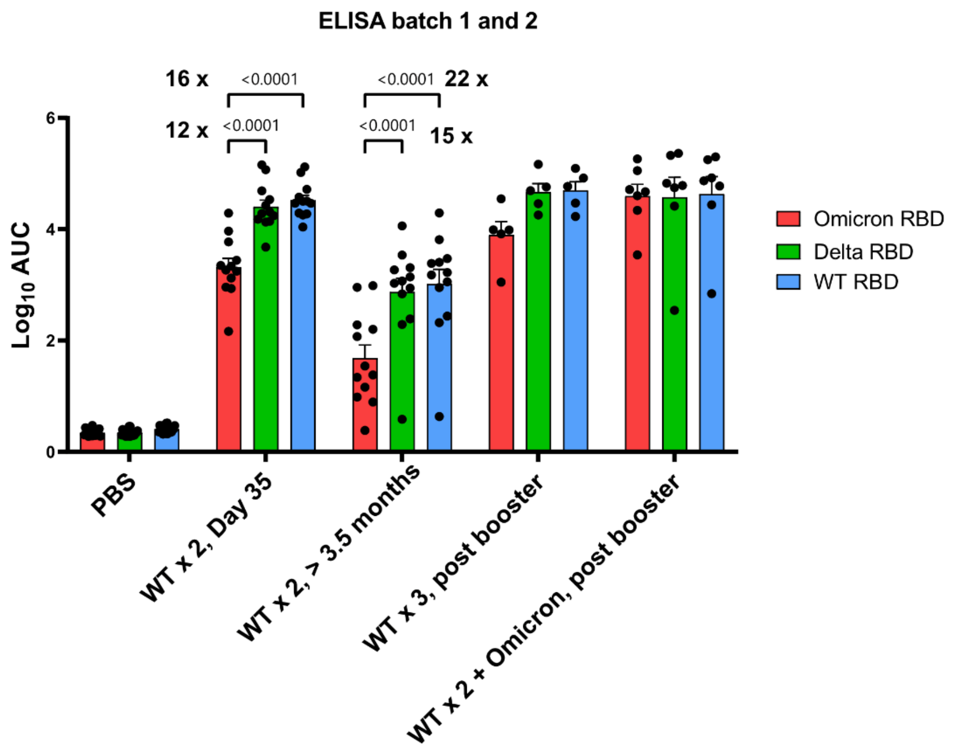

Supplementary Figure 5. Binding antibody titers of mice administered with PBS or WT and Omicron LNP-mRNA against Omicron, Delta and WT RBD (ELISA antigens), were grouped by vaccination timepoints to compare titers against different RBD antigens. The antibody titers were quantified as area under curve of $\log_{10}$-transformed titration curve (Log_{10} AUC) in Extended Data Figure 2. The data were derived from independent experiment 1 and 2.

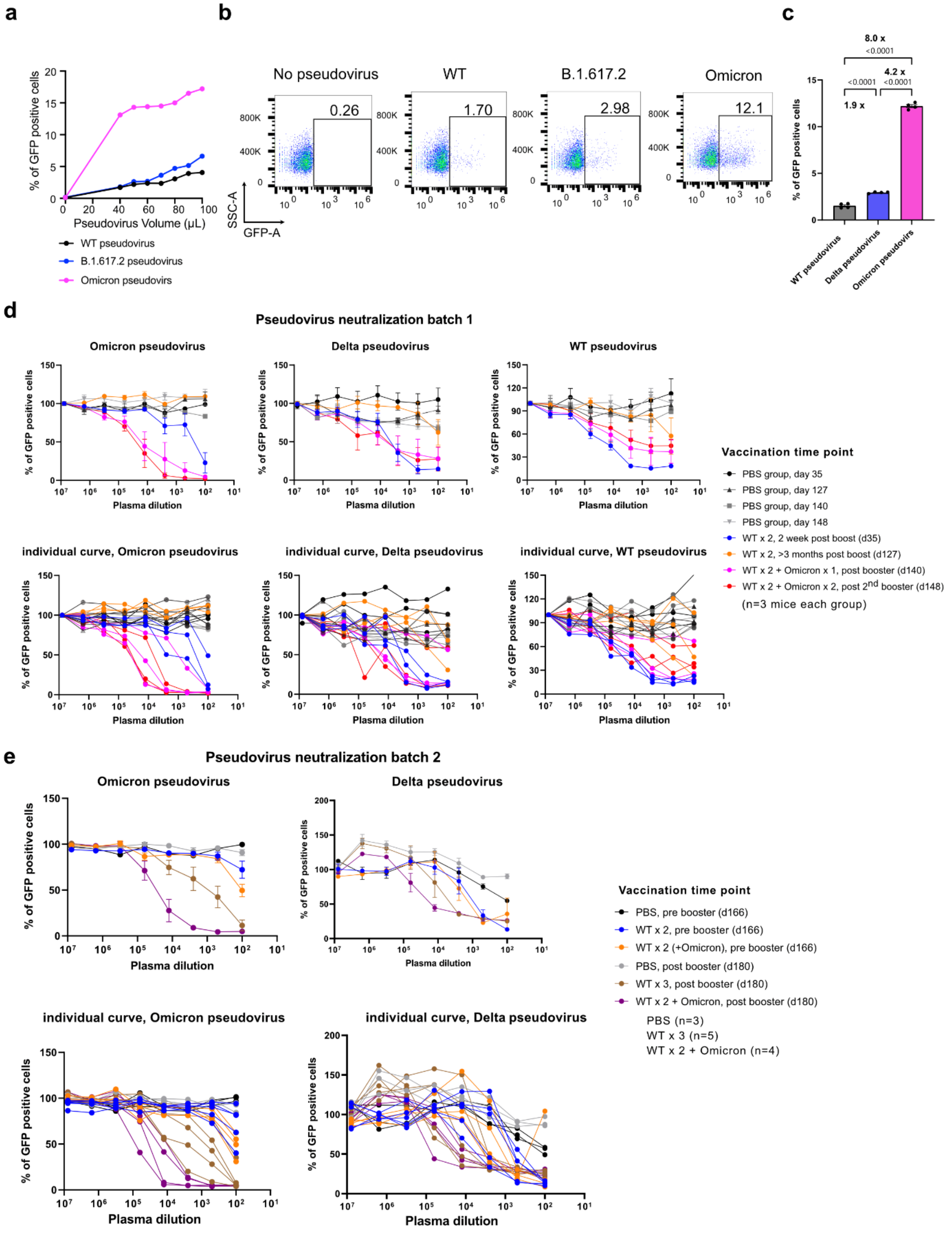
a
% of GFP positive cells
Pseudovirus Volume (μL)
WT pseudovirus
B.1.617.2 pseudovirus
Omicron pseudovirs
b
No pseudovirus
WT
B.1.617.2
Omicron
0.26
1.70
2.98
12.1
SSC-A
GFP-A
c
8.0 x
<0.0001
4.2 x
1.9 x
WT pseudovirus
Delta pseudovirus
Omicron pseudovirs
d
Pseudovirus neutralization batch 1
Omicron pseudovirus
Delta pseudovirus
WT pseudovirus
individual curve, Omicron pseudovirus
individual curve, Delta pseudovirus
individual curve, WT pseudovirus
Plasma dilution
Vaccination time point
PBS group, day 35
PBS group, day 127
PBS group, day 140
PBS group, day 148
WT x 2, 2 week post boost (d35)
WT x 2, >3 months post boost (d127)
WT x 2 + Omicron x 1, post booster (d140)
WT x 2 + Omicron x 2, post 2nd booster (d148)
(n=3 mice each group)
e
Pseudovirus neutralization batch 2
Omicron pseudovirus
Delta pseudovirus
individual curve, Omicron pseudovirus
individual curve, Delta pseudovirus
Vaccination time point
PBS, pre booster (d166)
WT x 2, pre booster (d166)
WT x 2 (+Omicron), pre booster (d166)
PBS, post booster (d180)
WT x 3, post booster (d180)
WT x 2 + Omicron, post booster (d180)
PBS (n=3)
WT x 3 (n=5)
WT x 2 + Omicron (n=4)

Supplementary Figure 6. Omicron, Delta and WT pseudovirus production, characterization and neutralization assay.

a. Functional titration curves of Omicron, Delta and WA-1 pseudoviruses in hACE2+ cells.

b. Representative Flow Cytometry plots of infectivity of Omicron, Delta and WA-1 pseudoviruses in hACE2+ cells.

c. Quantification of infectivity of Omicron, Delta and WT pseudoviruses in hACE2+ cells (n = 4).

d, Neutralization titration curves from batch 1 experiment (n = 3).

e. Neutralization titration curves from batch 2 experiment (n = 9 before booster, n=5 in WT x 3, n = 4 in WT x 2 + Omicron).

Percent of pseudovirus infected cells was plotted over serial dilutions of plasma from mice 35 days post WT prime, >4 months post WT prime (day 127 in batch 1 and day 166 in batch 2) and 2 weeks post booster (day 140 in batch 1 and day 180 in batch 2) of WT and Omicron LNP-mRNA against Omicron (left), Delta (mid) and WT (right) pseudovirus. Pseudovirus infection rate was calculated from percent of GFP positive cells and was plotted against plasma dilution ($\log_{10}$ transformed) as titration curve. Top panels, average curves, data are shown as mean ± s.e.m.; Bottom panels, individual curves. Sample number is designated as n from biologically independent samples. One-way ANOVA with Holm-Sidak multiple comparisons test was used to assess statistical significance. Statistical significance labels: * $p < 0.05$; ** $p < 0.01$; *** $p < 0.001$; **** $p < 0.0001$.

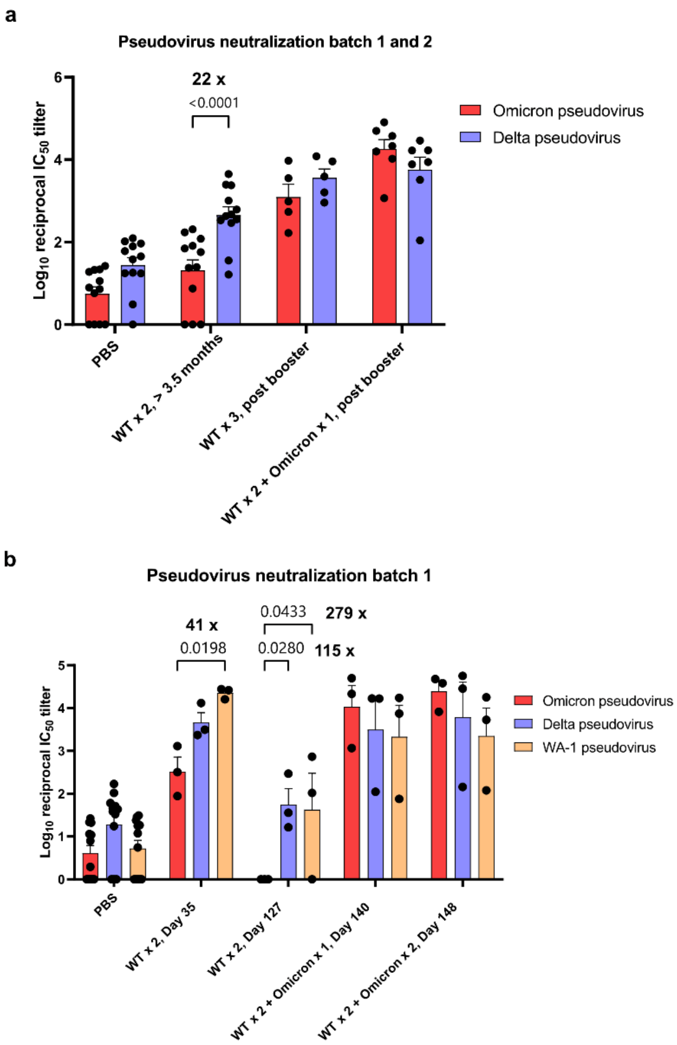

Supplementary Figure 7. Neutralizing antibody titers in the form of $\log_{10}$-transformed reciprocal IC50 were grouped by vaccination timepoints to compare titers against different pseudoviruses.

The neutralization titers from combined datasets (a) or batch 1 (b) were quantified as $\log_{10}$-transformed reciprocal IC50 values (Log_{10} reciprocal IC50, or Log_{10} IC50) based on titration curves in Supplementary Figure 6.

Titer ratios were indicated in each graph and fold change is calculated from (ratio - 1).

Data on dot-bar plots are shown as mean ± s.e.m. with individual data points in plots. Two-way ANOVA with Tukey's multiple comparisons test was used to assess statistical significance. Statistical significance labels: * $p < 0.05$; ** $p < 0.01$; *** $p < 0.001$; **** $p < 0.0001$. Non-significant comparisons are not shown, unless otherwise noted as n.s., not significant.

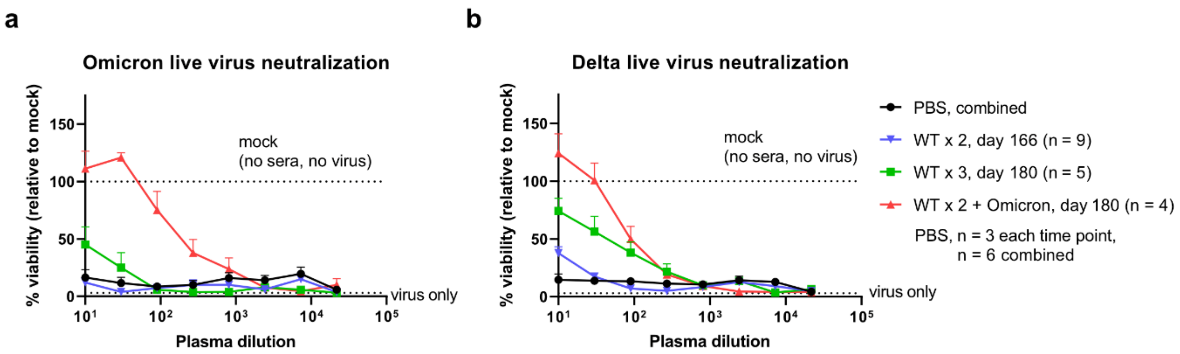

Supplementary Figure 8. Live virus neutralization titration curves over serial dilution of plasma collected at different timepoints from mice administered with PBS or WT and/or Omicron LNP-mRNA.

a, Omicron live virus titration curves (n = 9 before booster, n=5 in WT x 3, n = 4 in WT x 2 + Omicron)

b. Delta live virus titration curves (n = 9 before booster, n=5 in WT x 3, n = 4 in WT x 2 + Omicron) Titration curves were plotted over serial dilution points of plasma collected from mice before and after WT or Omicron LNP-mRNA boosters at defined time points. Data of each sample were collected from two replicates. Data are shown as mean + s.e.m. in plots.

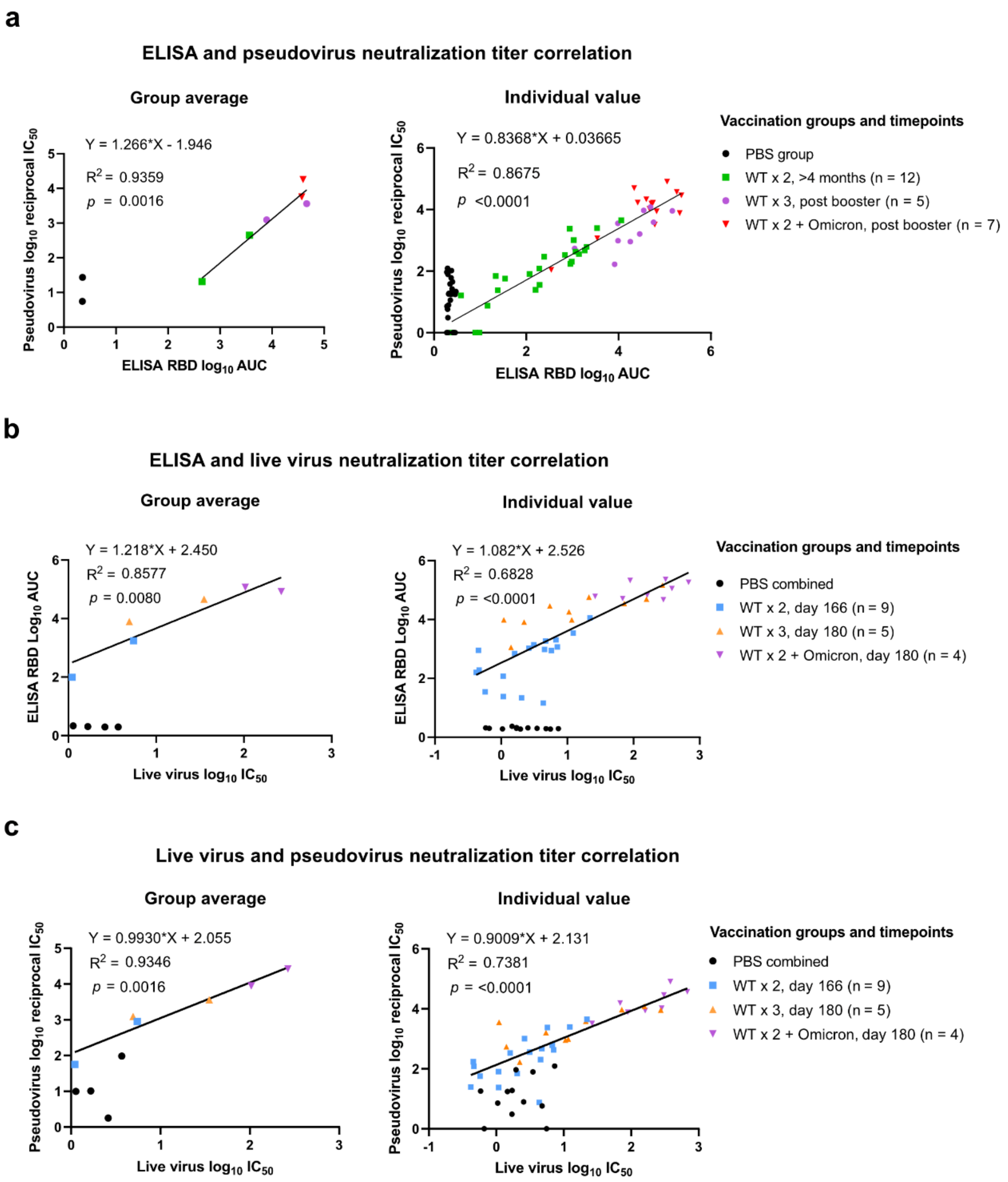
a
ELISA and pseudovirus neutralization titer correlation
Group average
Y = 1.266*X - 1.946
R^2 = 0.9359
p = 0.0016
Pseudovirus $\log_{10}$ reciprocal IC_{50}
ELISA RBD $\log_{10}$ AUC
Individual value
Y = 0.8368*X + 0.03665
R^2 = 0.8675
p <0.0001
Pseudovirus $\log_{10}$ reciprocal IC_{50}
ELISA RBD $\log_{10}$ AUC
Vaccination groups and timepoints
PBS group
WT x 2, >4 months (n = 12)
WT x 3, post booster (n = 5)
WT x 2 + Omicron, post booster (n = 7)
b
ELISA and live virus neutralization titer correlation
Group average
Y = 1.218*X + 2.450
R^2 = 0.8577
p = 0.0080
ELISA RBD $\log_{10}$ AUC
Live virus $\log_{10}$ IC_{50}
Individual value
Y = 1.082*X + 2.526
R^2 = 0.6828
p = <0.0001
ELISA RBD $\log_{10}$ AUC
Live virus $\log_{10}$ IC_{50}
Vaccination groups and timepoints
PBS combined
WT x 2, day 166 (n = 9)
WT x 3, day 180 (n = 5)
WT x 2 + Omicron, day 180 (n = 4)
c
Live virus and pseudovirus neutralization titer correlation
Group average
Y = 0.9930*X + 2.055
R^2 = 0.9346
p = 0.0016
Pseudovirus $\log_{10}$ reciprocal IC_{50}
Live virus $\log_{10}$ IC_{50}
Individual value
Y = 0.9009*X + 2.131
R^2 = 0.7381
p = <0.0001
Pseudovirus $\log_{10}$ reciprocal IC_{50}
Live virus $\log_{10}$ IC_{50}
Vaccination groups and timepoints
PBS combined
WT x 2, day 166 (n = 9)
WT x 3, day 180 (n = 5)
WT x 2 + Omicron, day 180 (n = 4)

Supplementary Figure 9. Correlation analysis of antibody titers determined by ELISA, pseudovirus neutralization and live virus neutralization assays.

a, Correlation between pseudovirus neutralization titers (log_{10} reciprocal IC50, y axis) and ELISA titers (log_{10} AUC, x axis) from matched vaccination group (left panel) or individual mouse (right panel).

b. Correlation between live virus neutralization titers (log_{10} IC50, x axis) and ELISA titers (log_{10} AUC, y axis) from matched vaccination group (left panel) or individual mouse (right panel).

c. Correlation between live virus neutralization titers (log_{10} IC50, x axis) and pseudovirus neutralization titers (log_{10} AUC, y axis) from matched vaccination group (left panel) or individual mouse (right panel).

PBS samples from different timepoints were shown as one group in correlation map and were not included in linear regression model. Each dot in bar graphs represents value from one group average (left panel), or one individual mouse (right panel). The Prism default two-side simple linear regression test (is slope significantly non-zero?) without multiple comparison adjustment was used to assess statistical significance.

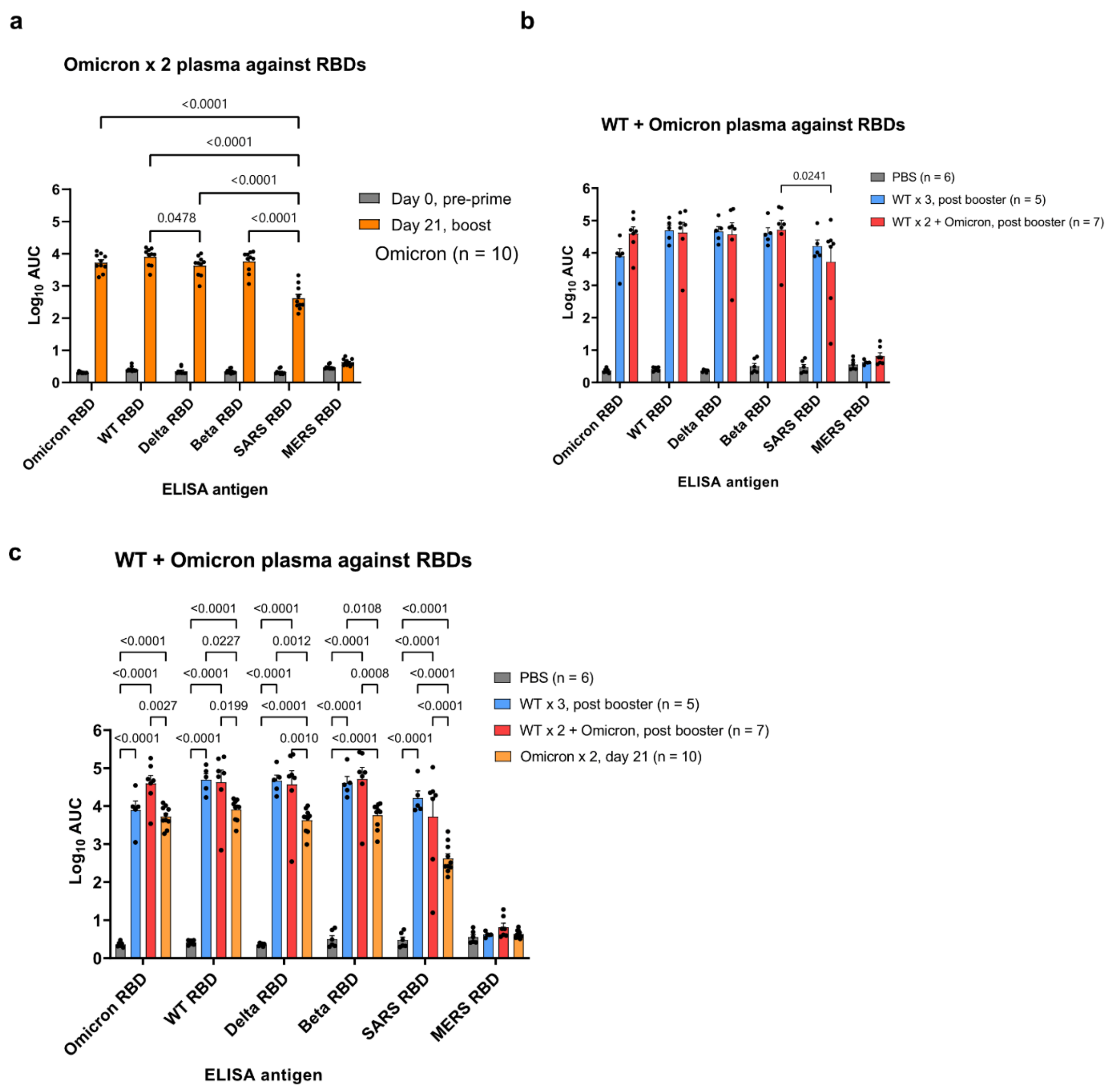

Supplementary Figure 10. Assessment of WT or Omicron LNP-mRNA mediated cross reactivity against a panel of SARS-CoV-2 variants and pathogenic coronavirus species in ELISA.

a, binding antibody titers (Log_{10} AUC) of plasma from mice that received Omicron LNP-mRNA prime and boost (Omicron x 2, n = 10).

b, binding antibody titers of plasma from mice that received WT (WT x 3, n = 5) or Omicron (WT x 2 + Omicron, n = 7) LNP-mRNA boosters.

c, binding antibody titers of plasma from mice that received Omicron LNP-mRNA prime + boost (Omicron x 2, n =10), WT (WT x 3, n = 5) or Omicron (WT x 2 + Omicron, n = 7) LNP-mRNA boosters.

This supplementary figure is a combination of data from the experiment shown in Figures 1 and 3 for comparison clarity.

Data on dot-bar plots are shown as mean ± s.e.m. with individual data points in plots. Two-way ANOVA with Tukey's multiple comparisons test was used to assess statistical significance. Multiple comparisons between titers against different ELISA antigens were made within same vaccination group. All comparisons with MERS RBD were significant and not shown in graph to simplify comparisons. Statistical significance labels: * $p < 0.05$; ** $p < 0.01$; *** $p < 0.001$; **** $p < 0.0001$. Non-significant comparisons are not shown.

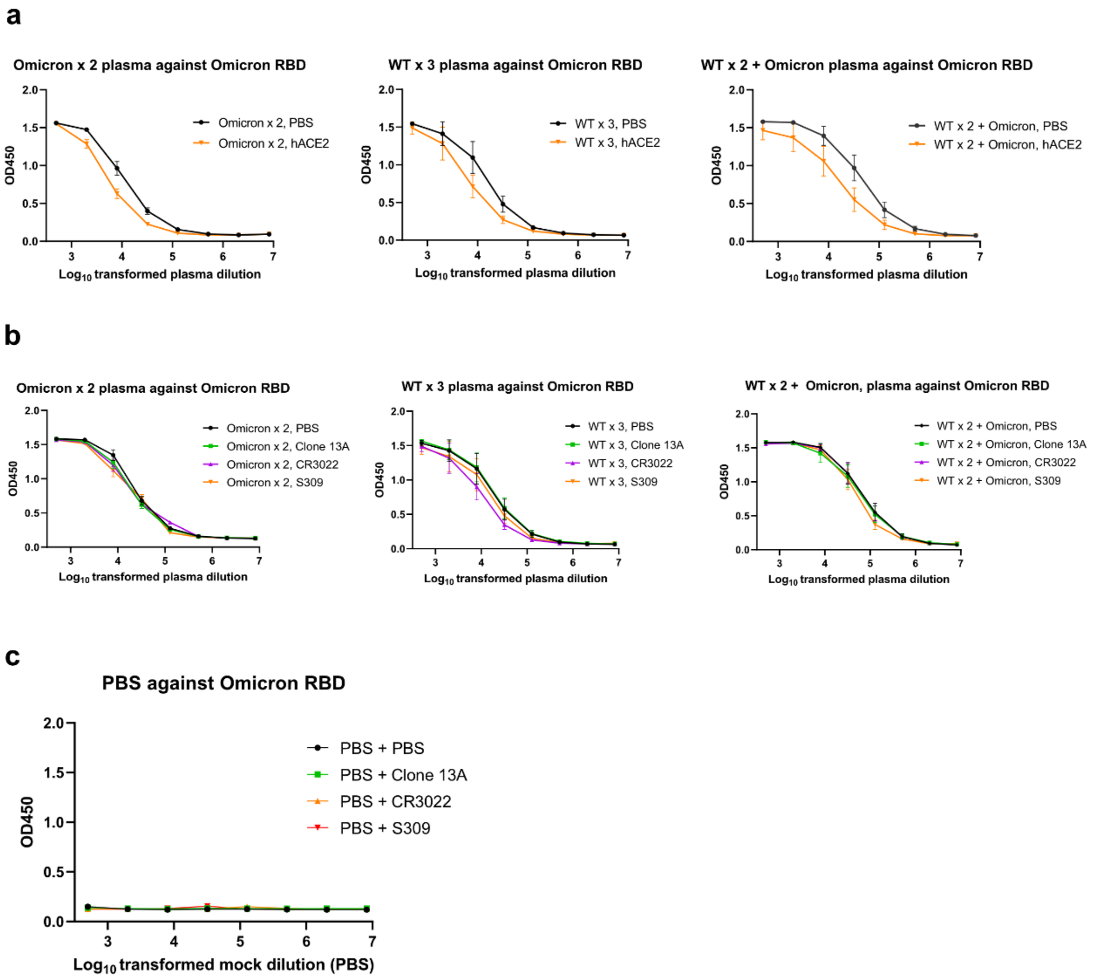

Supplementary Figure 11. Competition ELISA titration curves and binding antibody titers against low-density Omicron RBD from mice vaccinated with WT and/or Omicron LNP-mRNA.

a. hACE competition ELISA titration curves over a series of log_{10}-transformed dilution points of plasma from mice vaccinated with Omicron LNP-mRNA (Omicron x 2 plasma, left, n = 10) or WT/Omicron LNP-mRNA (WT x 3, middle, n = 5 and WT x 2 + Omicron plasma, right, n = 7).

b. antibody competition ELISA titration curves over a series of log_{10}-transformed dilution points of plasma from mice vaccinated with Omicron LNP-mRNA (Omicron x 2 plasma, left, n = 10) or WT/Omicron LNP-mRNA (WT x 3 plasma, middle, n = 5 and WT x 2 + Omicron plasma, right, n = 7).

c. PBS buffer as negative control to show minimal cross reactivity of anti-mouse secondary antibody with human IgG blocking antibodies, including Clone 13A, CR3022 and S309. n = 2 and each contains 8 mock dilution points.

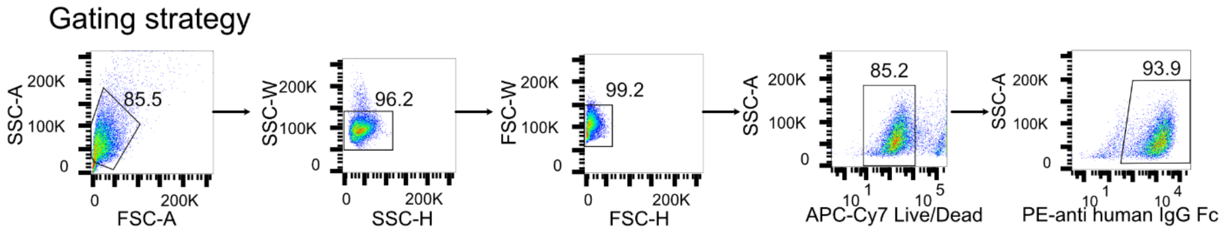

Supplementary Figure 12. Representative flow cytometry gating strategy for detecting Omicron spike binding to human ACE2 receptor.

Supplementary Table 1. List of oligos

46_gb0:
gtgggaggtctatataagcagagctctctggctaactagagaacccactgcttactggcttatcgaaattaatacgactcactatagggagacccaagctg
gctagccaccGAGAATAAACTAGTATTCTTCTGGTCCCCACAGACTCAGAGAGAACCCGCCACCA
TgTTTGTGTTCCTGGTGCTGCTGCCACTGGTGTCCAGCCAGTGTGTGAACCTGACCACCAGGA
CCCAACTTCCTCCTGCCTACACCAACTCCTTCACCAGGGGAGTCTACTACCCTGACAAGGTG
TTCAGGTCCTCTGTGCTGCACAGCACCCAGGACCTGTTCCTGCCATTCTTCAGCAATGTGACC
TGGTTCCATGTGATCTCTGGCACCAATGGCACCAAGAGGTTTGACAACCCTGTGCTGCCATT
CAATGATGGAGTCTACTTTGCCAGCAtcGAGAAGAGCAACATCATCAGGGGCTGGATTTTTGG
CACCACCCTGGACAGCAAGACCCAGTCCCTGCTGATTGTGAACAATGCCACCAATGTGGTGA
TTAAGGTGTGTGAGTTCCAGTTCTGTAATGACCCATTCCTGGacCACAAGAACAACAAGTCCT
GGATGG

46_gb1:

CACAAGAACAACAAGTCCTGGATGGAGTCTGAGTTCAGGGTCTACTCCTCTGCCAACAACTG
TACCTTTGAATATGTGAGCCAACCATTCCTGATGGACTTGGAGGGCAAGCAGGGCAACTTCA
AGAACCTGAGGGAGTTTGTGTTCAAGAACATTGATGGCTACTTCAAGATTTACAGCAAACAC
ACACCAATCatcGTGAGGGAGCCCGAGGACCTGCCACAGGGCTTCTCTGCCTTGGAACCACTG
GTGGACCTGCCAATTGGCATCAACATCACCAGGTTCCAGACCCTGCTGGCTCTGCACAGGTC
CTACCTGACACCTGGAGACTCCTCCTCTGGCTGGACAGCAGGAGCAGCAGCCTACTATGTGG
GCTACCTCCAACCAAGGACCTTCCTGCTGAAATACAATGAGAATGGCACCATCACAGATGCT
GTGGACTGTGCCCTGGACCCACTGTCTGAGACCAAGTGTACCCTGAAATCCTTCACAGTGGA
GAAGGGCATCTACCAGACCAGCAACTTCAGGGTCCAACCAACAGAGAGCATTGTGAGGTTT
CCAAACATCACCAACCTGTGTCCATTTGacGAGGTGTTCAAcGCCACCAGGTTTGCCTCTGTCT
ATGCCTGGAACAGG

46_gb2:

GCCTCTGTCTATGCCTGGAACAGGAAGAGGATTAGCAACTGTGTGGCTGACTACTCTGTGCT
CTACAACctgGCCcCCTTCttCACCTTCAAGTGTTATGGAGTGAGCCCAACCAAACTGAATGACC
TGTGTTTCACCAATGTCTATGCTGACTCCTTTGTGATTAGGGGAGATGAGGTGAGACAGATT
GCCCCTGGACAAACAGGCAAcATTGCTGACTACAACTACAAACTGCCTGATGACTTCACAGG
CTGTGTGATTGCCTGGAACAGCAACAAgCTGGACAGCAAGGTGagcGGCAACTACAACTACCT
CTACAGACTGTTCAGGAAGAGCAACCTGAAACCATTTGAGAGGGACATCAGCACAGAGATT
TACCAGGCTGGCAaCAagCCATGTAATGGAGTGGccGGCTTCAACTGTTACTTTCCACTCCggTC
CTATaGCTTCCggCCAACCtacGGAGTGGGCcACCAACCATACAGGGTGGTGGTGCTGTCCTTTG
AACTGCTCCATGCCCCTGCCACAGTGTGTGGACCAAAGAAGAGCACCAACCTGGTGAAGAA
CAAGTGTGTGAACTTCAACTTCAATGGACTGAagGGCACAGGAGTGCTGACAGAGAGCAACA
AGAAGTTCCTGCCATTCCAACAGTTTGGCAGGGACATTGCTGACACCACAGATGCTGTGAGG
GACCCACAGACCTTGGAGATTCTGGACATCACACCATGTTCCTTTGGAGGAGTGTCTGTGAT
TACACCTGGCACCAACACCAGCAACCAGGTGGCTGTGCTCTACCAGGgcGTGAACTGTACTG
AGGTGCCTGTGGCTATCCATGCTGACCAACTTACACCAACCTGGAGGGTCTACAGCACAGGC
AGCAATGTGTTCCAGACCAGGGCTGGCTGTCTGATTGGAGCAGAGtacGTGAACAACTCCTAT
GAGTGTGACATCCCAATTGGAGCAGGCATCTG

46_gb3:

CCCAATTGGAGCAGGCATCTGTGCCTCCTACCAGACCCAGACCAAgAGCCacGGCTCTGCATC
TTCTGTGGCAAGCCAGAGCATCATTGCCTACACAATGAGTCTGGGAGCAGAGAACTCTGTGG
CTTACAGCAACAACAGCATTGCCATCCCAACCAACTTCACCATCTCTGTGACCACAGAGATT
CTGCCTGTGAGTATGACCAAGACCTCTGTGGACTGTACAATGTATATCTGTGGAGACAGCAC
AGAGTGTAGCAACCTGCTGCTCCAATATGGCTCCTTCTGTACCCAACTTAAgAGGGCTCTGAC
AGGCATTGCTGTGGAACAGGACAAGAACACCCAGGAGGTGTTTGCCCAGGTGAAGCAGATT
TACAAGACACCTCCAATCAAGtACTTTGGAGGCTTCAACTTCAGCCAGATTCTGCCTGACCCA
AGCAAGCCAAGCAAGAGGTCCCCTATTGAGGACCTGCTGTTCAACAAGGTGACCCTGGCTG
ATGCTGGCTTCATCAAGCAATATGGAGACTGTCTGGGAGACATTGCTGCCAGGGACCTGATT
TGTGCCCAGAAGTTCAAgGGACTGACAGTGCTGCCTCCACTGCTGACAGATGAGATGATTGC
CCAATACACCTCTGCCCTGCTGGCTGGCACCATCACCTCTGGCTGGACCTTTGGAGCAGGAC
CAGCCCTCCAAATCCCATTTCCAATGCAGATGGCTTACAGGTTCAATGGCATTGGAGTGACC
CAGAATGTGCTCTATGAGAACCAGAAACTGATTGCCAACCAGTTCAACTCTGCCATTGGCAA
GATTCAGGACTCCCTGTCCAGCACACCATCTGCCCTGGGCAAACTCCAAGATGTGGTGAACC
AcAATGCCCAGGCTCTGAACACCCTGGTGAAGCAACTTTCCAGCAAgTTTGGAGCCATCTCCT
CTGTGCTGAATGACATCttcAGCAGACTGGACCCACCAGAGGCTGAGGTCCAGATTGACAGAC
TG

47_gb0:

cactatagggagacccaagctggctagccaccATgTTTGTGTTCCTGGTGCTGCTGCCACTGGTGTCCAGCCAGT
GTGTGAACCTGACCACCAGGACCCAACTTCCTCCTGCCTACACCAACTCCTTCACCAGGGGA
GTCTACTACCCTGACAAGGTGTTCAGGTCCTCTGTGCTGCACAGCACCCAGGACCTGTTCCT
GCCATTCTTCAGCAATGTGACCTGGTTCCATGTGATCTCTGGCACCAATGGCACCAAGAGGT
TTGACAACCCTGTGCTGCCATTCAATGATGGAGTCTACTTTGCCAGCAtcGAGAAGAGCAACA
TCATCAGGGGCTGGATTTTTGGCACCACCCTGGACAGCAAGACCCAGTCCCTGCTGATTGTG
AACAATGCCACCAATGTGGTGATTAAGGTGTGTGAGTTCCAGTTCTGTAATGACCCATTCCT
GGacCACAAGAACAACAAGTCCTGGATG

47_gb3:

CCCAATTGGAGCAGGCATCTGTGCCTCCTACCAGACCCAGACCAAgAGCCacAGGAGGGCAA
GGTCTGTGGCAAGCCAGAGCATCATTGCCTACACAATGAGTCTGGGAGCAGAGAACTCTGT
GGCTTACAGCAACAACAGCATTGCCATCCCAACCAACTTCACCATCTCTGTGACCACAGAGA
TTCTGCCTGTGAGTATGACCAAGACCTCTGTGGACTGTACAATGTATATCTGTGGAGACAGC
ACAGAGTGTAGCAACCTGCTGCTCCAATATGGCTCCTTCTGTACCCAACTTAAgAGGGCTCTG
ACAGGCATTGCTGTGGAACAGGACAAGAACACCCAGGAGGTGTTTGCCCAGGTGAAGCAGA
TTTACAAGACACCTCCAATCAAGtACTTTGGAGGCTTCAACTTCAGCCAGATTCTGCCTGACC
CAAGCAAGCCAAGCAAGAGGTCCTTCATTGAGGACCTGCTGTTCAACAAGGTGACCCTGGCT
GATGCTGGCTTCATCAAGCAATATGGAGACTGTCTGGGAGACATTGCTGCCAGGGACCTGAT
TTGTGCCCAGAAGTTCAAgGGACTGACAGTGCTGCCTCCACTGCTGACAGATGAGATGATTG
CCCAATACACCTCTGCCCTGCTGGCTGGCACCATCACCTCTGGCTGGACCTTTGGAGCAGGA

GCAGCCCTCCAAATCCCATTTGCTATGCAGATGGCTTACAGGTTCAATGGCATTGGAGTGAC
CCAGAATGTGCTCTATGAGAACCAGAAACTGATTGCCAACCAGTTCAACTCTGCCATTGGCA
AGATTCAGGACTCCCTGTCCAGCACAGCCTCTGCCCTGGGCAAACTCCAAGATGTGGTGAAC
CAcAATGCCCAGGCTCTGAACACCCTGGTGAAGCAACTTTCCAGCAAgTTTGGAGCCATCTCC
TCTGTGCTGAATGACATCttcAGCAGACTGGACAAGGTGGAGGCTGAGGTCCAGATTGACAGA
CTG

CMV-F:

CGCAAATGGGCGGTAGGCGTG

47_F1:

GGGCTTCTCTGCCTTGGAACCAC

47_F2:

GGCAACAAGCCATGTAATGGAGTG

47_F3:

CTACACAATGAGTCTGGGAGCAGAG
