# Supplementary material for: Omicron-specific mRNA vaccination alone and as a heterologous booster against SARS-CoV-2
Source: Nat Commun. 2022 Jun 6;13:3250. doi: 10.1038/s41467-022-30878-4 (PMC9169595; doi:10.1038/s41467-022-30878-4)
Supplement: Supplementary file 3 — Reporting Summary [file 41467_2022_30878_MOESM3_ESM.pdf]

nature portfolio

Corresponding author(s): Sidi Chen, Craig B. Wilen

Last updated by author(s): 5/28/2022

Reporting Summary

Nature Portfolio wishes to improve the reproducibility of the work that we publish. This form provides structure for consistency and transparency in reporting. For further information on Nature Portfolio policies, see our Editorial Policies and the Editorial Policy Checklist.

Statistics

For all statistical analyses, confirm that the following items are present in the figure legend, table legend, main text, or Methods section.

n/a	Confirmed	
☐	☒	The exact sample size (n) for each experimental group/condition, given as a discrete number and unit of measurement
☐	☒	A statement on whether measurements were taken from distinct samples or whether the same sample was measured repeatedly
☐	☒	The statistical test(s) used AND whether they are one- or two-sided<br>*Only common tests should be described solely by name; describe more complex techniques in the Methods section.*
☐	☒	A description of all covariates tested
☐	☒	A description of any assumptions or corrections, such as tests of normality and adjustment for multiple comparisons
☐	☒	A full description of the statistical parameters including central tendency (e.g. means) or other basic estimates (e.g. regression coefficient) AND variation (e.g. standard deviation) or associated estimates of uncertainty (e.g. confidence intervals)
☐	☒	For null hypothesis testing, the test statistic (e.g. F, t, r) with confidence intervals, effect sizes, degrees of freedom and P value noted<br>*Give P values as exact values whenever suitable.*
☒	☐	For Bayesian analysis, information on the choice of priors and Markov chain Monte Carlo settings
☐	☒	For hierarchical and complex designs, identification of the appropriate level for tests and full reporting of outcomes
☐	☒	Estimates of effect sizes (e.g. Cohen's d, Pearson's r), indicating how they were calculated

Our web collection on statistics for biologists contains articles on many of the points above.

Software and code

Policy information about availability of computer code

Data collection	Default softwares in the data collection instruments including Attune focusing cytometer (Attune NxT Software v3.1) and PerkinElmer EnVision 2105 microplate reader (Envision Manager v1.13.3009.1401) were used to collect data.
Data analysis	FlowJo (version 10.7.2, FlowJo LLC) for FACS, Prism (version 9.3.1, GraphPad Software Inc.) and RStudio (version 1.3.959, RStudio software company) for small scale data analysis.

For manuscripts utilizing custom algorithms or software that are central to the research but not yet described in published literature, software must be made available to editors and reviewers. We strongly encourage code deposition in a community repository (e.g. GitHub). See the Nature Portfolio guidelines for submitting code & software for further information.

Data

Policy information about availability of data

All manuscripts must include a data availability statement. This statement should provide the following information, where applicable:

- Accession codes, unique identifiers, or web links for publicly available datasets
- A description of any restrictions on data availability
- For clinical datasets or third party data, please ensure that the statement adheres to our policy

All data generated or analyzed during this study are included in this article and its supplementary information files. Specifically, source data and statistics are provided in a supplementary table excel file. No custom code was used in this study. Sequence of the Omicron variant (lineage B.1.1.529/BA.1) was derived from two North America patients in GISAID EpiCoV database with accession code of EPI_ISL_6826713 and EPI_ISL_6826714. Additional information related to this study are available from corresponding authors upon reasonable request.

Field-specific reporting

Please select the one below that is the best fit for your research. If you are not sure, read the appropriate sections before making your selection.

☒ Life sciences ☐ Behavioural & social sciences ☐ Ecological, evolutionary & environmental sciences

For a reference copy of the document with all sections, see nature.com/documents/nr-reporting-summary-flat.pdf

Life sciences study design

All studies must disclose on these points even when the disclosure is negative.

Sample size	For most cases, at least biological triplicate experiments (n>=3) were performed unless otherwise noted. Details on sample size for experiments were indicated in methods and figure legends. The sample size was based on the total number of mice immunized with WT LNP-mRNA 4 months prior the beginning of this study and also referenced similar work in the past (Peng, 2022, Nature Communications, https://www.nature.com/articles/s41467-022-29288-3#MOESM5).
Data exclusions	No data was excluded in this study.
Replication	Key animal immunization experiments in Figure 1 and 2 were replicated. The infectious virus neutralization assay were replicated. All replication experiments were successful and the findings were reproduced.
Randomization	Each mice was randomly allocated into experimental or control groups.
Blinding	Because of the need of sequential vaccination (especially for heterologous boosting) and limited personnel involved in animal study, the experiments were not blinded.

Reporting for specific materials, systems and methods

We require information from authors about some types of materials, experimental systems and methods used in many studies. Here, indicate whether each material, system or method listed is relevant to your study. If you are not sure if a list item applies to your research, read the appropriate section before selecting a response.

Materials & experimental systems

n/a	Involved in the study	
☐	☒	Antibodies
☐	☒	Eukaryotic cell lines
☒	☐	Palaeontology and archaeology
☐	☒	Animals and other organisms
☒	☐	Human research participants
☒	☐	Clinical data
☒	☐	Dual use research of concern

Methods

n/a	Involved in the study	
☒	☐	ChIP-seq
☐	☒	Flow cytometry
☒	☐	MRI-based neuroimaging

Antibodies

Antibodies used	1. S309 (Biovision, Cat. No. A2266, Clone No. S309, 10ug/ml final concentration)<br>2. CR3022 (Abcam, Cat. No. ab273073, Clone No. CR3022, 10ug/ml final concentration)<br>3. Clone 13A (produced by Chen lab, 10ug/ml final concentration)<br>4. Anti-mouse Fc secondary antibody with minimal cross reactivity with human IgG (Biolegend, Cat. No. 405306, Clone No. Poly4053, 1:2500 dilution)<br>5. PE–anti-human FC antibody (Biolegend, Cat. No. 410708, Clone No. M1310G05, 1:100 dilution)<br>6. HRP-conjugated anti-mouse Fc antibody (Fisher, Cat# A-10677, 1:2500 dilution)
Validation	Antibodies were validated by the vendors and re-validated through subsequent experiments. Custom and commercial antibodies were validated by specific antibody - antigen interaction assays, such as ELISA. Commercial antibody info and validation info where applicable:<br>1. S309 antibody (validated by ELISA and SDS-PAGE): https://www.biovision.com/anti-sars-cov-2-s1-antibody-clone-s309.html<br>2. CR3022 antibody (validated by ELISA): https://www.abcam.com/sars-cov-2-spike-glycoprotein-s1-antibody-cr3022-ab273073.html<br>3. Clone 13A validated by Chen lab's publication: https://www.nature.com/articles/s41467-022-29288-3<br>4. Anti-mouse secondary antibody (cross validated by 46 product citations in technical data sheet): https://www.biolegend.com/fr-lu/products/hrp-goat-anti-mouse-igg-minimal-x-reactivity-1395<br>5. PE-anti-human Fc antibody (validated by two product citations): https://www.biolegend.com/it-it/products/pe-anti-human-igg-fc-11933<br>6. HRP-conjugated anti-mouse Fc antibody (validated by product references): https://www.thermofisher.com/antibody/product/

Goat-anti-Mouse-IgG-IgM-H-L-Secondary-Antibody-Polyclonal/A-10677

Eukaryotic cell lines

Policy information about cell lines

Cell line source(s)	All commercial cell lines were originally acquired from commercial vendors (ATCC, ThermoFisher). HEK293T (ATCC CRL-3216), HEK293FT (Thermo Fisher R70007), Vero-E6 over-expressing ACE2/TMPRSS2 (Dr. Craig Wilen's lab) and 293T-hACE2 (gifted from Dr Bieniasz' lab) cell lines were used in this study.
Authentication	Cell lines were authenticated by original vendors, and re-validated in lab as appropriate by morphology, SARS-CoV-2 infection susceptibility (Vero-E6 over-expressing ACE2/TMPRSS2 and 293T-hACE2) and antibiotic resistance (stable cell lines, 293FT and 293T-hACE2).
Mycoplasma contamination	All cell lines tested negative for mycoplasma contamination.
Commonly misidentified lines (See ICLAC register)	No misidenified cell lines were used in the study.

Animals and other organisms

Policy information about studies involving animals; ARRIVE guidelines recommended for reporting animal research

Laboratory animals	C57BL/6Ncr (B6) female 6-8 week mice purchased from Charles River
Wild animals	The study did not involve in wild animals.
Field-collected samples	The study did not involve samples collected from the field.
Ethics oversight	All animal work was performed under the guidelines of Yale University Institutional Animal Care and Use Committee (IACUC) with approved protocols (Chen-2020-20358; Chen 2021-20068; Wilen 2021-20198).

Note that full information on the approval of the study protocol must also be provided in the manuscript.

Flow Cytometry

Plots

Confirm that:

- [x] The axis labels state the marker and fluorochrome used (e.g. CD4-FITC).
- [x] The axis scales are clearly visible. Include numbers along axes only for bottom left plot of group (a 'group' is an analysis of identical markers).
- [x] All plots are contour plots with outliers or pseudocolor plots.
- [x] A numerical value for number of cells or percentage (with statistics) is provided.

Methodology

Sample preparation	Various sample prep details in the Methods section
Instrument	Flow cytometry data were acquired by BD FACSAria cytometer (BD Bioscences)
Software	FlowJo software (version 10.7.2, FlowJo LLC) was used for flow cytometry data analysis.
Cell population abundance	Live cells with uniform size on flow cytometry were gated (approximately 70% of total population based on Figure S12) and analyzed for surface spike expression (~65% of total population).
Gating strategy	Cells were gated by FSC/SSC plot. To distinguish between positive and negative boundaries of the stained cells, negative control samples were analyzed and utilized as background.

- [x] Tick this box to confirm that a figure exemplifying the gating strategy is provided in the Supplementary Information.
